# Nogo receptor is involved in the adhesion of dendritic cells to myelin

**DOI:** 10.1186/1742-2094-8-113

**Published:** 2011-09-09

**Authors:** Claire L McDonald, Karin Steinbach, Florian Kern, Rüdiger Schweigreiter, Roland Martin, Christine E Bandtlow, Markus Reindl

**Affiliations:** 1 Clinical Department of Neurology, Innsbruck Medical University, Anichstrasse 35, A-6020 Innsbruck, Austria; 2 Centre for Molecular Neurobiology, Institute for Neuroimmunology and Clinical MS Research (inims), Falkenried 94, D-20251 Hamburg, Germany; 3 Division of Neurobiochemistry, Innsbruck Medical University, Biocenter, Fritz-Pregl-Strasse 3, A-6020 Innsbruck, Austria; 4 Department of Clinical Neuroimmunology and MS Research, Neurology Clinic, University Hospital Zürich, Frauenklinikstrasse 26, CH-8091 Zürich, Switzerland

**Keywords:** Nogo receptor, NgR1, NgR2, Nogo-66, myelin associated glycoprotein, MAG, myelin, dendritic cells

## Abstract

**Background:**

Nogo-66 receptor NgR1 and its structural homologue NgR2 are binding proteins for a number of myelin-associated inhibitory factors. After neuronal injury, these inhibitory factors are responsible for preventing axonal outgrowth via their interactions with NgR1 and NgR2 expressed on neurons. *In vitro*, cells expressing NgR1/2 are inhibited from adhering to and spreading on a myelin substrate. Neuronal injury also results in the presence of dendritic cells (DCs) in the central nervous system, where they can come into contact with myelin debris. The exact mechanisms of interaction of immune cells with CNS myelin are, however, poorly understood.

**Methods:**

Human DCs were differentiated from peripheral blood monocytes and mouse DCs were differentiated from wild type and NgR1/NgR2 double knockout bone marrow precursors. NgR1 and NgR2 expression were determined with quantitative real time PCR and immunoblot, and adhesion of cells to myelin was quantified.

**Results:**

We demonstrate that human immature myeloid DCs express NgR1 and NgR2, which are then down-regulated upon maturation. Human mature DCs also adhere to a much higher extent to a myelin substrate than immature DCs. We observe the same effect when the cells are plated on Nogo-66-His (binding peptide for NgR1), but not on control proteins. Mature DCs taken from *Ngr1/2 *knockout mice adhere to a much higher extent to myelin compared to wild type mouse DCs. In addition, *Ngr1/2 *knockout had no effect on *in vitro *DC differentiation or phenotype.

**Conclusions:**

These results indicate that a lack of NgR1/2 expression promotes the adhesion of DCs to myelin. This interaction could be important in neuroinflammatory disorders such as multiple sclerosis in which peripheral immune cells come into contact with myelin debris.

## Background

Injury to the central nervous system (CNS) has long been known to cause fatal and irreversible damage to axons and neurons. A number of physical and molecular inhibitory factors expressed by neurons, astrocytes, and oligodendrocytes serve to maintain the architecture of the mature CNS, but at the same time contribute to the lack of repair mechanisms following damage. Some of the major molecular inhibitors to regeneration are those associated with myelin (myelin-associated inhibitory factors, MAIFs). MAIFs include Nogo-A [1,2], myelin-associated glycoprotein (MAG) [3,4] and oligodendrocyte-myelin glycoprotein (OMgp) [5]. These factors are all binding partners for the Nogo-66 receptor-1 (NgR1), a mainly neuron-expressed, GPI-anchored protein [6-8]. Nogo-66 is a 66 amino acid long region of Nogo-A that binds NgR1 and is largely responsible for inhibiting neurite outgrowth. Since the identification of NgR1, two structural homologues have been discovered, termed NgR2 and NgR3. NgR2 is a high affinity binding protein for MAG [9,10] and the binding protein of NgR3 has not yet been identified. As NgR1 is a GPI-anchored protein, it requires co-receptors in order to transmit its signal inside the cell. Thus, it is often found assembled in a heterotrimeric complex composed of p75^NTR ^[7] or TROY [11], and LINGO-1 (Leucine rich repeat and Ig domain-containing, Nogo receptor-interacting protein) [12]. However, due to the findings of NgR1 expression without LINGO-1 [13], or without both TROY and p75^NTR ^[14], it is likely that more signal transducing subunits of the NgR1 complex remain to be identified. Binding of the NgR1 inhibitory complex by MAIFs leads to activation of intracellular RhoA, thereby resulting in axonal outgrowth inhibition, or modulation of cell adhesion and motility [15].

NgR1 expression has been identified in a few non-neuronal cell types, where it mediates adhesion of these cells to MAIFs. For example, fibroblasts, glioma cells, macrophages, and some human immune cells have all been found to express NgR1 and to be inhibited from adhering to myelin substrates [13,16-18]. Our aim was to expand on this data and to further clarify the role of NgRs in human immune cells. In this paper we focus on dendritic cells (DCs) due to their importance in a number of neuroinflammatory situations and due to the high NgR1 expression we found in immature DCs. DCs in the immature state are tissue resident and are responsible for surveying the tissue for possible insults. Upon activation by defined factors (cytokines, bacterial or viral molecules), DCs become mature and travel to lymph nodes to present antigen to T cells [19]. This change in function is reflected in the up-regulation of the antigen presenting molecules HLA-DR, CD86 and CD83, as well as the chemokine receptor CCR7 to aid cellular migration.

DCs are usually not present in the healthy brain, however, they have been found to accumulate in the CNS parenchyma during a wide range of inflammatory insults [20-22] and they are emerging as important players in CNS autoimmunity, specifically in multiple sclerosis (MS) [23]. Indeed, mature DC markers have been consistently found in the inflamed meninges and perivascular cuffs of most active MS lesions examined [24]. Thus, it would be valuable to further understand the role of DCs within the inflammatory milieu of CNS myelin debris.

In the current study, we demonstrate that NgR1 and NgR2 (referred to jointly as NgR1/2) are expressed to a higher extent by human immature myeloid DCs (immDCs) compared to mature myeloid DCs (matDCs). DCs that do not express NgR1/2 are more adherent when plated on a myelin substrate compared to those that express NgR1/2. Promotion of adhesion could also be demonstrated in mouse DCs genetically lacking NgR1/2. The interaction of DCs with myelin debris proposed here could have important implications for our understanding of how immune cells act within CNS inflammatory lesions.

## Methods

### Generation of human monocyte-derived dendritic cells

Whole human blood was obtained by venous puncture into EDTA tubes with informed, written consent from 9 healthy donors with approval from the local institutional review board of Innsbruck Medical University. Myeloid DCs were generated according to established standard procedures [25,26]. Firstly, peripheral blood mononuclear cells (PBMCs) were isolated from the blood by density gradient centrifugation using Ficoll™-based lymphocyte separation medium (PAA, Pasching, Austria). PBMCs were washed with 0.9% saline solution (Fresenius Kabi, Graz, Austria) and seeded at a density of 3.3 × 10^6 ^cells/ml in a 6-well plate in serum-free medium (Lonza x-vivo chemically-defined medium, Cologne, Germany). After two hours of incubation at 37°C, with 5% CO_2_, monocytes selectively adhered to the cell culture-treated plastic. At this stage, all non-adherent cell populations were washed away by rinsing three times with RPMI1640 medium (Gibco, Invitrogen, Carlsbad, CA, USA). After the washing steps, adherent monocytes were cultured for 8 days in serum-free medium supplemented with 1% penicillin streptomycin (PenStrep, Invitrogen, Carlsbad, CA, USA), 800 U/ml granulocyte/monocyte colony stimulating factor (GM-CSF, Novartis, Leukomax, Basel, Switzerland) and 40 ng/ml interleukin-4 (human recombinant IL-4, Invitrogen). Every two days, cells were fed with fresh medium, PenStrep, GM-CSF and IL-4. By day 6, the human monocytes had differentiated into loosely adherent immature dendritic cells (immDCs). Addition of a defined maturation cocktail for the last two days of culture resulted in generation of mature DCs (matDCs). Maturation cocktail (MC) consisted of interleukin 1β (2 ng/ml, Invitrogen), IL-6 (10 ng/ml, Invitrogen), tumour necrosis factor-α (TNF-α, 10 ng/ml, Invitrogen) and prostaglandin E2 (PGE2, 1 μg/ml, Sigma-Aldrich, St. Louis, MO, USA). On day 8 of culture, immature and mature DCs were harvested for flow cytometric analysis, RNA extraction, and adhesion assay.

### Isolation of human immune cell subsets

T cells were isolated from human PBMCs using a commercially available magnetic cell separator T cell depletion kit (Miltenyi Biotec GmbH, Bergisch Gladbach, Germany) to produce *ex vivo *T cells (Tex). Cells were cultured in serum-free medium in the presence of anti-CD3 antibody (T cont), and in the presence or absence of T cell activator phytohaemagglutinin (T PHA+ and T PHA-, respectively) for 2 days. Epstein Barr virus-transformed B lymphocytes were used as a B cell line (BCL). Monocytes were isolated from PBMCs by magnetic cell separator monocyte depletion kit (Miltenyi Biotec GmbH) to produce *ex vivo *monocytes. They were then maintained in serum-free medium for 7 days, and given either interferon gamma (IFN-γ, 100 ng/ml, Invitrogen) or lipopolysaccharide (LPS, 100 ng/ml, Sigma-Aldrich) for the last two days of culture.

### Generation of mouse bone marrow-derived dendritic cells

Wild type male C57BL/6J mice were obtained from Jackson Laboratories and housed in the animal house of Innsbruck Medical University. *Ngr1/2 *double knockout mice (*Ngr1/2-/-*) were generated by crossing *Ngr1-/- *mice [27] with *Ngr2-/- *mice as previously described [10]. Bone marrow derived myeloid DCs were prepared according to established standard procedures as described by Lutz et al. [28]. Mice were sacrificed by cervical dislocation and the tibiae and femurs were removed. The bones were cleaned of all muscle tissue and sterilised with 70% ethanol. The bone marrow was flushed out with cold RPMI1640 containing 10% foetal calf serum (FCS, Sigma-Aldrich) and β-mercaptoethanol (β-ME, 50 μM, Sigma-Aldrich). The marrow was separated into a single cell suspension by repeated pipetting and passed through a nylon mesh to remove bone and debris. Contaminating erythrocytes were removed by lysis on ice using erythrocyte lysis buffer (containing 0.15 M ammonium chloride, 10 mM potassium bicarbonate, 0.1 mM EDTA (all from Roth, Karlsruhe, Germany), with pH adjusted to 7.0-7.2) and cells were counted. 20 × 10^6 ^bone marrow precursor cells were seeded in RPMI containing 10% FCS, 50 μM β-ME, and 20 ng/ml GM-CSF (ImmunoTools, Friesoythe, Germany) in 75 cm^3 ^flasks. After two days, flasks were gently swirled and 75% of medium was removed. The same volume of fresh medium was added back, containing 40 ng/ml GM-CSF. On day 4, the culture is made up of firmly attached stromal cells covered in clusters of loosely attached DCs, and non-adherent granulocytes. The granulocytes were washed away and DCs were subcultured at a concentration of 1 × 10^6 ^cells/ml/well in a 24-well plate, with 20 ng/ml GM-CSF. On day 6, cells were fed by removal of 75% of the medium and adding back the same volume containing GM-CSF. For the generation of mature DCs, maturation cocktail containing a final concentration of 2 ng/ml IL-1β (Invitrogen), 10 ng/ml IL-6 (ImmunoTools), 10 ng/ml TNF-α (ImmunoTools), and 1 μg/ml PGE-2 (Sigma-Aldrich) was added for the last two days of culture. Cells were harvested for flow cytometric analysis, RNA extraction, and adhesion assay on day 8.

### Flow cytometric analysis

In order to define and compare the phenotype of *in vitro*-generated human and mouse DCs, cells were characterised by flow cytometry. Briefly, cells were washed with FACS Cell Wash solution (Becton Dickinson Biosciences, San Jose, CA, USA) and 200000 cells in 100 μl Cell Wash solution were used per staining. Each staining consisted of a fluorescein isothiocyanate (FITC)-labelled antibody and phycoerythrin (PE)-labelled antibody, occasionally in combination with a peridinin chlorophyll protein complex (perCP)-labelled antibody. The following fluorescently labelled antibodies were used for detection of human DC antigens: HLA-DR-PerCP (BD Biosciences), CD86-FITC (BD Biosciences), CCR7-PE (R&D Systems, Minneapolis, MN, USA), CD83-FITC (BD Biosciences), CD11b-PE (BD Biosciences), CD1a-FITC (BD Biosciences), and CD11c-PE (BioLegend, San Diego, CA, USA). Fluorescently labelled mouse antibodies were: MHC II-PE (BD Biosciences), CD86-FITC (BioLegend), CCR7-PE (BioLegend), CD83-FITC (BioLegend), CD11b-PE (BioLegend), CD11c-PE (BioLegend), and CD14-FITC (BD Biosciences). First, human or mouse DCs were blocked for 15 minutes with 2 μg/200000 cells human or mouse IgG, respectively. Fluorescently labelled antibodies were added to the cells at the concentration suggested by the manufacturer, and incubated for 20 minutes at room temperature in the dark. Cells were washed and resuspended in 300 μl Cell Wash before being analysed by flow cytometry with a BD FACScan instrument using Cell Quest Pro Software (BD Biosciences).

### Determination of mouse supernatant cytokine concentrations

The following cytokines were measured in cell culture supernatant from mouse immature and mature DCs from WT and *Ngr1/2-/- *mice: 6Ckine, CTACK, Eotaxin, GCSF, GM-CSF, IL-2, IL-3, IL-4, IL-5, IL-9, IL-10, IL-12p40p70, IL-12p70, IL-13, IL-17, IFN-γ, KC, Leptin, MCP-1, MCP-5, MIP-1α, MIP-2, MIP-3β, RANTES, SCF, sTNFRI, TARC, TIMP-1, Thrombopoietin, and VEGF. Cytokine levels were determined as per the protocol using the Ray Biotech mouse cytokine antibody array G2 (AAM-CYT-G2-8, RayBiotech, Norcross, GA, USA). The array consists of antibody-coated glass slides that were pre-treated according to the manufacturer's instructions and incubated with cell culture supernatants for 2 hours. All sample measurements were performed in duplicate. The glass slides were then washed, incubated with a biotin-conjugated anti-cytokine mix for 2 hours, washed again, and developed for 2 hours with Cy3-conjugated streptavidin. The arrays were scanned for fluorescent signals with a GenePix 4000B scanner (Axon Instruments, GenePix version 5.0) and analysed with the Ray biotech analysis tool, a data analysis program based on Microsoft Excel technology specifically designed to analyse Ray biotech G Series Antibody Arrays. Signals were normalised using positive and negative controls included on the array.

### RNA isolation and real time quantitative PCR

Cells (a minimum of 10^6^) were washed with PBS and homogenised in 1 ml TRIzol reagent (Invitrogen). RNA was extracted as per the manufacturer's protocol, dissolved in diethylpyrocarbonate (DEPC)-treated water and the concentration was determined using a spectrophotometer (NanoDrop 1000, peqlab, Polling, Austria). 1 μg of RNA from each sample was reverse transcribed with the High Capacity cDNA Reverse Transcription Kit (Applied Biosystems, Carlsbad, CA, USA) using random primers. The protocol was followed as described in the kit. cDNA was diluted 1:4 and immediately used for RT qPCR. Levels of Nogo receptor component mRNAs in human and mouse DCs were determined using TaqMan RT qPCR Assays (Applied Biosystems). Assays used for human mRNA detection were: NgR1 (Hs00368533_m1), NgR2 (Hs00604888_m1), LINGO-1 (Hs01072978_m1), TROY (Hs00218634_m1) and p75^NTR ^(Hs00609977_m1). Assays used for mouse mRNA detection were as follows: NgR1 (Mm00452228_m1), NgR2 (Mm01336368_g1), LINGO-1 (Mm01173306_m1), TROY (Mm00443506_m1), and p75^NTR ^(Mm00446296_m1). 18 s rRNA was measured in each sample as an endogenous control in order to control for varying cDNA concentrations and human or mouse brain cDNA were used as a positive control for all assays (commercially available human foetal brain RNA was used from Clontech Laboratories, Inc., Mountain View, CA, USA). Assays were performed as described by the manufacturer, with a final assay volume of 25 μl. Experiments were performed in duplicate wells and all assays were first screened for detection of genomic DNA. Data were collected using the 7300 Real-Time PCR System (Applied Biosystems) and analysed by the comparative Ct method, where; ΔCt = target Ct - endogenous Ct; and ΔΔCt = ΔCt_matDC _- ΔCt_immDC_; relative mRNA expression = 2^-ΔΔCt^. Immature DCs were assigned as the calibrator for all relative quantifications, except where otherwise stated.

### Western Blot

Human brain and human immature and mature DCs were lysed in buffer containing 150 mM NaCl, 1% Triton X-100, 10% glycerol, 50 mM Hepes pH 7.40, and protease inhibitor cocktail (Roche Applied Sciences, Mannheim, Germany). Protein concentration of all lysates was determined with the bicinchoninic acid protein assay (BCA, Sigma-Aldrich). 22 μg of human brain, and 70 μg each of immDC and matDC protein were denatured and loaded onto a 10% Bis/Tris gel (Invitrogen). After separation, protein was blotted onto a Hybond membrane (Amersham, GE Healthcare, Buckinghamshire, UK) and probed with NgR11-A antibody (diluted 1:3000, Alpha Diagnostic, San Antonio, TX, USA). Detection was performed with horseradish peroxidase-conjugated secondary antibodies and enhanced chemiluminescence detection on film (Amersham, GE Healthcare). To confirm antibody specificity, NgR11-A was first blocked with the immunising peptide (NgR11-P, Alpha Diagnostic) at 5 times the weight of NgR11-A used. In order to probe the same membranes for actin, they were stripped at 60°C with buffer containing 2% SDS, 100 mM beta-mercaptoethanol and 62.5 mM Tris-HCl, pH 6.8, then washed, blocked and incubated with anti-actin monoclonal antibody (diluted 1:20000, BD Biosciences).

### Myelin extraction from brain

Myelin was isolated from central nervous system tissue by the density gradient centrifugation method, as described previously [29]. Briefly, a segment of human brain, or whole mouse CNS was shock frozen in liquid nitrogen and stored at -80°C until needed. Tissue was thawed on ice and cut into smaller pieces and homogenised in a 0.32 M sucrose solution. The homogenized tissue was washed three times in 0.32 M sucrose before being layered over a 0.85 M sucrose solution. After centrifugation at 26000 × g for 60 minutes at 4°C, myelin was contained in the interphase between the high and low sucrose solutions. The myelin was subjected to osmotic shock by stirring with distilled water for 30 minutes at 4°C, before being washed and ultracentrifuged again with 0.32 M sucrose. Finally, the myelin was washed three times with, and resuspended in distilled water. The protein concentration of the myelin extract was determined with BCA protein assay.

### MBP extraction

Human myelin derived MBP was purified from normal human brain according to the procedure of Eylar et al. [30]. SDS-PAGE and Western blot with a monoclonal antibody to MBP 130-137 (Millipore GmbH, Vienna, Austria) was used to confirm the purity of the MBP preparation.

### Cloning and production of recombinant proteins

A DNA fragment encoding the mouse Nogo-66 loop was amplified from the mouse Nogo-A clone IRAVp968A04133D (ImaGene, Berlin, Germany) with the primers mM_RTN4-66-s (5'-CTA CCA TGG GCA GGA TAT ATA AGG GTG TGA TCC-3') and mM_RTN4-66-as (5'-GCT TGC GGC ACC CTT CAG GGA ATC AAC TAA ATC-3'). The fragment was digested with NcoI and ligated into pET28a(+) vector using the NcoI and a blunted NotI site. The sequence was verified by sequencing at LGC Genomics (Berlin).

E. coli Rosetta were transformed with Nogo-66 pET28 and induced in 1 litre of LB culture medium with 1 mM IPTG at an OD600 of 0.6 and a temperature of 30°C. After 3 hours the bacteria were harvested by centrifugation at 4000 × g for 5 minutes. The pellet was resuspended in PBS substituted with 1% Triton X-100 and protease inhibitors and sonicated. After 30 minutes of incubation on ice the lysate was centrifuged and the pellet dissolved in 8 M urea. Recombinant Nogo-66-His was purified with TALON^®^Cobalt resin (Clontech Laboratories) under denaturing conditions and eluted with elution buffer (50 mM Hepes pH 4, 300 mM imidazol, 150 mM NaCl). Finally, the eluate was dialysed against DMEM adjusted to pH 4. Protein concentration was determined by BCA assay (Thermo Scientific, Rockford, IL, USA).

MAG-Fc was produced as described previously [31]. Briefly, conditioned medium of transiently transfected CHO-K1 cells was harvested and recombinant protein was purified using Protein A/G Agarose (Thermo Scientific, Rockford, IL, USA). Purity and concentration were confirmed by comparing band intensity on SDS-PAGE to BSA standard.

### DC adhesion assay

Adhesion assays for human DCs were conducted in 96-well plates. All cell types and conditions were assayed in triplicate. The following substrates were all used at concentrations of 100 and 10 μg/ml for adhesion assay: human myelin, human MBP, His-tagged mouse Nogo-66 (Nogo-66-His, amino acids 1025-1090). 50 μl of each substrate was added to the 96-well plate and incubated for 4 hours at 37°C. MAG-Fc was added at 10 μg/ml and was coated on human IgG to aid clustering of the protein. First, 15 μg/ml human IgG (Sigma-Aldrich) was added to wells in 50 mM bicarbonate buffer (pH 9) and incubated over night at 4°C. The next day, wells were washed and MAG-Fc was added along with the other adhesion substrates to their respective wells and the plate was incubated for 4 hours at 37°C, 5% CO_2_. Excess substrate was removed and all wells were washed once with medium before addition of cells. Human monocyte-derived DCs were prepared as described above and harvested on day 8. Cells were collected, counted and plated at 200000 cells/ml in the 96-well plate in serum-free medium. Cells were allowed to adhere for 30 minutes at 37°C, 5% CO_2_. Non-adherent cells were gently removed and wells were washed three times with medium. In order to detect and count CD11b^+ ^DCs, cells were fixed and fluorescently stained as follows. Cells were fixed with 4% paraformaldehyde (PFA) for 30 minutes at room temperature. After the PFA was washed away, nonspecific antibody binding was blocked by addition of 20 μg/ml human IgG in PBS containing 5% normal goat serum (NGS, Invitrogen) and 1% bovine serum albumin (BSA, Sigma-Aldrich) for 1 hour at room temperature. In order to detect adherent DCs, CD11b antibody (BD Biosciences) diluted 1:100 in 1% NGS, 1% BSA was added to the cells and incubated over night at 4°C with gentle shaking. The following day, the cells were washed three times with PBS and visualised at 10 × magnification with a fluorescent microscope (Leica Microsystems, Cambridge, UK). Four digital photos were taken per well and cells were counted using the particle analysis tool from ImageJ [32].

Adhesion of mouse DCs was assayed in 96-well plates and based on the method described by Kueng et al [33]. 10 μg/ml mouse myelin was added to the respective wells and incubated for 4 hours at 37°C. Myelin was removed and wells were washed once with medium before addition of cells. Mouse bone marrow-derived DCs were prepared as described above and harvested on day 8. Cells were collected, counted and plated at 200000 cells/ml in triplicate in the 96-well plate in RPMI 1640. Cells were allowed to adhere for 30 minutes at 37°C, 5% CO_2_. Non-adherent cells were gently removed and wells were washed three times with medium. In order to quantify the number of remaining adherent cells, they were stained with cresyl violet and absorbance was measured as follows. Cells were fixed with 4% PFA for 30 minutes at room temperature. Cells were stained with 0.04% cresyl violet (Sigma-Aldrich) in 20% methanol for 30 minutes. The dye was then extracted with 0.1 M citric acid in 50% ethanol for 30 minutes on a rotating shaker. Absorbance of each well was measured at 570 nm using the DTX 880 Multimode Detector with Multimode Analysis Software (Beckman Coulter, Krefeld, Germany).

### Statistical analysis

All statistical analyses were carried out using GraphPad Prism 5 software (GraphPad Software Inc., San Diego, CA, USA). For flow cytometry data, an unpaired, two-tailed student's t test was used to compare the means of each marker in immature versus mature DCs. Microarray data was analysed using TIGR MeV_4_5 (Multiple Experiment Viewer), a Java tool for genomic data analysis http://mev-tm4.sourceforge.net[34] which measures significance of microarray (SAM). Multi-class SAM was used to identify significant cytokines based on differential expression between the four groups at a false discovery rate (FDR, proportion of genes likely to have been identified by chance as being significant) of 0%. To determine significance of RT qPCR data, ΔCt values were compared using the Wilcoxon matched-pairs signed rank test, as per Yuan et al. [35]. Human DC adhesion to myelin was measured using a two-way repeated measure ANOVA. The association of human RT qPCR ΔCt values with adhesion was calculated using Spearman correlation. Mouse immature WT vs. *Ngr1/2-/- *and mature WT vs. *Ngr1/2-/- *adhesion to myelin were analysed using the Wilcoxon matched-pairs signed rank test.

## Results

### Expression of NgRs in human and mouse DCs

**Figure 1:**
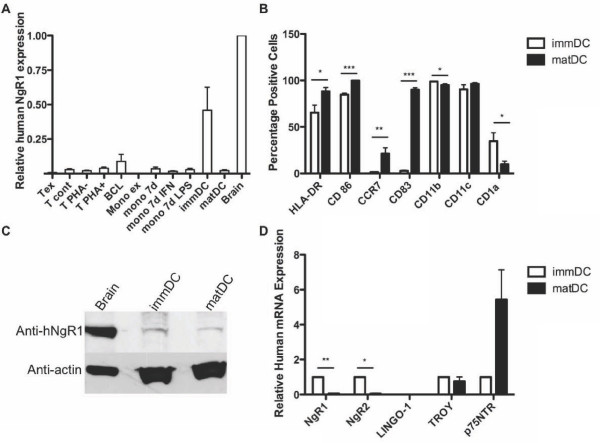
**NgR expression in human immune cells**. (**A**) Expression of NgR1 mRNA in a panel of human immune cells, as determined by TaqMan RT qPCR. Expression relative to human foetal brain is depicted. Tex: *ex vivo *T cells; T cont: T cells cultured with anti-CD3 antibody for 2 days; T PHA-: T cells cultured with anti-CD3 and without PHA; T PHA+: T cells cultured with anti-CD3 and stimulated with PHA; BCL: B cell line; Mono ex: *ex vivo *monocytes; mono 7d: monocytes maintained in serum-free medium for 7 days; mono 7d IFN: 7d mono's treated with interferon gamma for the last 2 days; mono 7d LPS: 7d mono's treated with lipopolysaccharide for the last 2 days; Brain: commercially available human foetal brain RNA. (**B**) Expression of cell surface markers on human monocyte-derived DCs, quantified with flow cytometry. Bars represent mean of percentage of positive cells for the indicated marker, with SEM of 8 experiments, each representing a different donor. *P < 0.05, **P < 0.01, ***P < 0.001. (**C**) A representative western blot of NgR1 protein expression in human brain, immature and mature DC. (**D**) Relative mRNA expression of NgR1, NgR2, LINGO-1, TROY and p75^NTR ^were determined with TaqMan RT qPCR. Mean values relative to immDC with SEM from 8 donors are shown. Wilcoxon matched-pairs signed rank test was used with delta Ct values to determine significance. *P < 0.05, **P < 0.01.

As our aim was to expand on current knowledge of the role of NgRs in non-CNS cells, we began the study as a screen for NgR1 expression in human peripheral immune cells. Expression of NgR1 mRNA was measured in a panel of human immune cells (un-stimulated and stimulated T cells, B cell line, monocytes, immature and mature DCs) using TaqMan real time quantitative PCR (RT qPCR). Expression of NgR1 mRNA was five times higher in immature DCs compared to all other immune cells tested (Figure 1A). We thus concentrated on further examining NgR expression in DCs. Human monocyte derived myeloid DCs were generated as described and first characterised by flow cytometry (Figure 1B). More than 95% of the cells expressed CD11b, indicating high purity of monocyte-derived DCs. The DC phenotype was confirmed by high CD11 c expression (Figure 1B). Only 2.8 ± 0.6% of untreated DCs expressed CD83. The percentage of cells expressing CD83 increased significantly to 90.0 ± 2.2% after addition of maturation cocktail, indicating successful DC maturation. Significant increases in expression of human leukocyte antigen (HLA), CD86, CCR7 and a decrease in CD1a provide further evidence of successful maturation (Figure 1B). Having verified the *in vitro *generation of myeloid DCs, we went on to examine the regulation of NgR expression in these cells.

NgR1 expression was increased in comparison to the monocytes from which the immDCs were generated (Figure 1A). It is then down-regulated upon maturation. The increased transcription was confirmed by higher protein expression of NgR1 in human immature DCs, as determined by western blot (Figure 1C). This regulation of NgR1 expression between immature and mature DCs prompted us to also measure the expression of NgR1's co-receptors, as well as of NgR2. NgR2 mRNA is down-regulated in the same manner upon maturation of human DCs (Figure 1D). Expression of NgR1 co-receptors LINGO-1, TROY and p75^NTR ^was not down-regulated upon maturation in the same way as NgR1.

**Figure 2:**
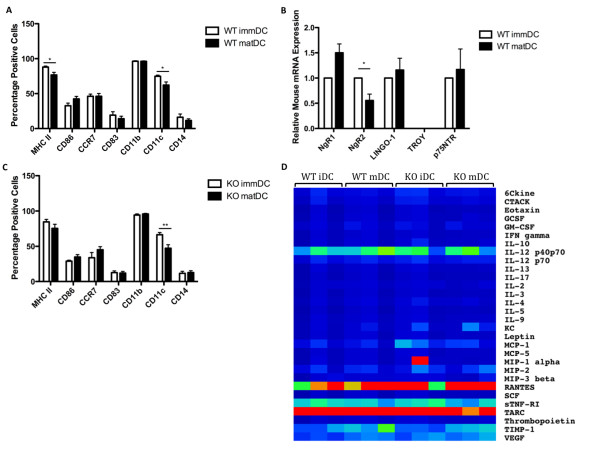
**Mouse WT and *Ngr1/2-/- *DC characterisation and NgR expression**. Expression of cell surface markers on mouse WT **(A) **and *Ngr1/2-/- *(KO) **(C) **bone marrow derived DCs, as quantified with flow cytometry. Bars represent mean of percentage of positive cells for the indicated marker, with SEM of at least 6 experiments (6 of each WT and *Ngr1/2-/- *mice). *P < 0.05, **P < 0.01. (**B**) Relative mRNA expression of NgR1, NgR2, LINGO-1, TROY and p75^NTR ^were determined with TaqMan RT qPCR. Mean values relative to immDC with SEM from 9 experiments, representing 9 mice, are shown. Wilcoxon matched-pairs signed rank test was used with delta Ct values to determine significance. *P < 0.05, **P < 0.01. (**D**) A panel of 32 cytokines were measured in cell culture supernatant from 3 mice of each: WT immDC (WT iDC) and matDC (WT mDC), and *Ngr1/2-/- *immDC (KO iDC) and matDC (KO mDC). The relative concentrations (as a ratio to the positive control of the assay) of these cytokines are shown as a heatmap. Low concentrations are shown in blue, median concentrations in green and high concentrations in red.

Due to the fact that we later used mouse DCs in our functional analysis of *Ngr1/2 *knockout, we also characterised mouse bone marrow derived DCs and analysed expression of NgR1, NgR2 and co-receptors. The characterisation of WT mouse DC is shown in Figure 2A. Like human DC, more than 95% of mouse *in vitro*-generated DCs express CD11b, suggesting a high purity of monocyte-derived DCs, whereas CD14 expression was low. There is a trend towards higher CD86 and lower CD11 c upon maturation, in concurrence with Lutz et al. [28].

In contrast to human DCs, mouse NgR1 expression does not change significantly upon maturation; however there is a trend towards up-regulation in mature DCs (Figure 2B). NgR2 expression on the other hand, is significantly down-regulated upon addition of maturation cocktail, however not to the same extent as in human mature DCs. LINGO-1 and p75^NTR ^are expressed at similar levels as NgR1 in mouse immature and mature DCs but also do not follow the same expression pattern observed in human DCs. Discrepancies in expression of NgRs between human and mouse DCs are also reflected in expression of DC cell surface markers, suggesting we are not dealing with directly comparable cell types. Furthermore, in both the human and mouse systems, we did not observe NgR1's co-receptors being regulated in the same way as NgR1. However, as it has previously been demonstrated that NgR1 can function without the full complement of identified co-receptors [13,14,36], we went on to determine the functional role of NgR1 and NgR2 in human and mouse DCs.

### Myelin promotes adhesion of DCs lacking NgR1 and NgR2 expression

**Figure 3:**
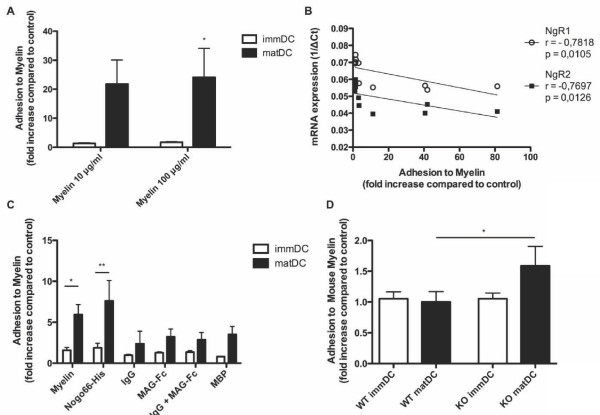
**Adhesion of human and mouse DCs to myelin**. (**A**) Adhesion of human DCs (8 healthy donors) to human myelin. Values were calculated as the fold change in adhesion on myelin compared to adhesion of the same cells to plastic control (plastic = 1). (**B**) Human immature and mature DC (grouped) NgR1 and NgR2 mRNA expression (expressed as 1/delta Ct, 1/ΔCt) correlate with adhesion to a myelin substrate (at 100 μg/ml), expressed as the fold increase in adhesion on myelin compared to plastic. NgR1, Spearman r = -0.7818, *P *= 0.0105. NgR2, Spearman r = -0.7697, *P *= 0.0126. (**C**) Adhesion of human DCs (3 donors) to myelin, Nogo-66-His, MAG-Fc, MBP (all at 10 μg/ml), IgG (15 μg/ml), expressed as fold increase in adhesion compared to on plastic. (**D**) Adhesion of mouse WT and *Ngr1/2-/- *(KO) DCs (9 mice of each genotype) to mouse myelin (10 μg/ml), expressed as fold increase in adhesion to myelin compared to plastic. Bars represent mean with SEM of the fold change in adhesion on myelin compared to adhesion of the same cells to plastic (plastic = 1). *P < 0.05, **P < 0.01.

NgR1/2 have been found to mediate the inhibition of cellular adhesion to myelin. Thus, in order to determine the possible function of NgR1/2 down-regulation in human mature DCs, adhesion of immature and mature DCs to a myelin substrate was quantified. Immature and mature DCs were plated on human myelin and adhesion was then calculated as the fold change in adhesion compared to on plastic control. Human mature DCs were found to adhere significantly more to human CNS myelin compared to immature DCs (Figure 3A). As mentioned above, human mature DCs down-regulate NgR1 and NgR2 (Figure 1D). Figure 3B depicts the correlation between NgR1/2 mRNA expression (graphed as the raw value for both, 1/ΔCt) in all DCs and their adhesion to myelin, showing that increased NgR1/2 expression correlates significantly with decreased adhesion to myelin.

In order to identify which protein fraction of myelin is responsible for promoting adhesion of matDCs, we isolated various components of myelin that do or do not interact with NgRs, and measured adhesion of the cells in comparison to plastic control. His-tagged Nogo-66 recombinant peptide was plated at the same concentrations as myelin and used as a positive control for NgR1. As a positive control for NgR2, MAG-Fc was used. As MAG-Fc needs to be clustered in order to function correctly, the plate was first coated with IgG and then MAG-Fc was added. Myelin basic protein (MBP) was isolated from human brain white matter and used as a negative control as it is not known to bind or activate NgR1 or NgR2. Nogo-66-His demonstrated the same effect on adhesion of immature and mature DC as seen with myelin (Figure 3C). That is, mature DCs (which express less NgR1/2) were found to adhere to a much higher extent to Nogo-66-His than immature DCs. Adhesion of mature DCs to MBP and MAG-Fc was found to remain at a background level and not reach statistical significance. To ensure that the adhesion observed with Nogo-66-His was not due to side effects of the His-tag or bacterial contamination, we performed a control adhesion assay with two His-tagged and bacterially expressed peptides that do not bind NgR1/2. Neither NiR-His (amino acids 1 - 172 of rat Nogo-A) nor the 66-amino acid loop domain of RTN1 had any effect on adhesion of DCs (data not shown), thus suggesting that increased adhesion of matDCs to Nogo-66-His is indeed specific. This confirms that Nogo contained in the myelin preparation mediates promotion of matDC adhesion. Generally, matDCs adhere better to any substrate, however, the increase is most significant for myelin and Nogo-66-His. This indicates that Nogo might mediate this effect (probably due to the loss of NgR1).

Having found that Nogo-66 promotes adhesion of human matDC, we wanted to further clarify if it is indeed the loss of NgR1 expression that is the functional cause for increased adhesion of matDCs to myelin. To this end, we took DCs from NgR1/2 double knockout (*Ngr1/2-/-*) mice and compared them to wild type (WT) before measuring how they adhere to a myelin substrate. The double knockout mice were used rather than the single knockouts in order to exclude an effect resulting from the possible compensatory up-regulation of NgR2 in *Ngr1-/- *DCs.

DCs generated *in vitro *from WT and *Ngr1/2-/- *mice show similar phenotypes (Figure 2A and 2C). Furthermore, 32 cytokines released from WT and *Ngr1/2-/- *DCs were compared in cell culture supernatants using a glass chip protein array system (Figure 2D). We found no significant changes in secreted cytokines from mouse WT and *Ngr1/2-/- *DCs, thus indicating that the deletion of NgR1 and NgR2 had no influence on the differentiation and phenotype of DC. However, in the adhesion assay we did observe that mature DCs from *Ngr1/2-/- *mice adhere significantly more to myelin than mature DCs from WT mice (p = 0.02, Figure 3D). This indicates that a lack of NgR1/2 in mouse mature DCs promotes their adhesion to a myelin substrate.

## Discussion

We describe here the enhanced adhesion of human mature DCs to human CNS myelin, and that this enhanced adhesion is mediated by a down-regulation of NgR1 expression. We propose that high NgR1 expression in human immature DCs prevents their adhesion to a myelin substrate and that reduced NgR1 expression in mature DCs promotes the adhesion of those cells to myelin.

Previous studies on NgR1/2 expression in immune cells have also shown that where NgR1/2 are expressed, there is an inhibition of adhesion to myelin. This was shown in a rat peripheral nerve lesion model in which macrophages invading the lesion site began to express NgR1 and NgR2 7 days after injury [18]. At this stage, the macrophages were inhibited from adhering to myelin and to MAG, and indeed were found to migrate away from the lesion site as soon as healthy myelin began to regenerate. This effect was not observed both in MAG knockout mice and when NgR1/2 were down-regulated in macrophages with siRNA [18]. As peripheral nervous system myelin contains higher concentrations of MAG and very little Nogo, it is most likely the interaction of MAG with NgR2 that is being described. Another publication to describe NgR1 expression in immune cells demonstrates that NgR1 is up-regulated in activated human T cells *in vitro *and that these cells show a reduced adhesion to myelin [13]. However, this effect was shown to be unaffected by the NgR1-specific antagonist NEP1-40. DCs were also not analysed as part of this study. We were able to advance these findings by using highly sensitive TaqMan RT qPCR to measure regulation of NgR1 gene expression in human DCs. Although the expression of NgR1's identified co-receptors was not regulated in the same way as NgR1, we went on to study the functional relevance of NgR1 expression in human DCs. This is due to previous findings of functioning NgR1 in the absence of LINGO-1 and/or p75^NTR ^and TROY [11,13,14,37], which leads us to suggest that there are as yet unidentified co-receptors which can act as the signal transducing subunit of the NgR1 complex. We went on to conclusively demonstrate that in human matDCs, which lack NgR1 expression, there is an increased adhesion to myelin. This is supported by our demonstration of increased adhesion to myelin of mouse matDC genetically lacking NgR1/2.

Taking a closer look at adhesion of mouse WT DCs to myelin, we see a marked difference in how they adhere to myelin when compared with human DCs. These cells also show different patterns of expression of NgR1/2 and co-receptors. Furthermore, when comparing the expression of the various DC surface markers, it becomes obvious that human and mouse *in vitro *generated DCs demonstrate phenotypical differences. An explanation for the variation between the two species could be that the cells undergo distinct differentiation procedures. As mentioned, mouse DCs are differentiated directly from precursor cells present in the bone marrow. Human DCs, on the other hand, are differentiated from blood-borne monocytes. This variation in preparation could lead to differences in phenotype of this highly heterogeneous cell family. A number of publications have also addressed the issue of dissimilarities not only between human and mouse DCs but also in the functions of the various populations of DCs found *in vivo *when compared to those that are generated *in vitro *[38-40]. In both humans and mice, several DC subsets have been identified based on differences in phenotypes, anatomical locations or functions [41,42]. These subsets are generated *in vivo *with very complex and specific environmental influences, which have not yet been replicated in culture. Thus, both the different experimental preparations of DCs and endogenous inter-species variation could contribute to the observed variations in cell types.

Our results further suggest that NgR1 and/or NgR2 may not play such a significant role in DC interaction with myelin in the mouse WT system. This is in line with the observation of no change in immune response after EAE was induced in *Ngr1/2-/- *mice, as well as no difference in the number of CNS invading DCs after EAE was induced in *Ngr1/2-/- *mice compared to WT [43].

The described regulation of human NgR1/2 expression could have a number of implications for DCs both during the normal immune response and in autoimmune diseases. The results presented here show a decrease in NgR1 and NgR2 expression upon maturation of human DCs. Immature DCs have been well described as phagocytic cells expressing low levels of chemokine receptors. Upon maturation, DCs are no longer phagocytic, they up-regulate the chemokine receptor CCR7 and are highly migratory [44]. The complex processes of matDC migration involve adhesion to and transmigration across a number of different cell types and extracellular matrices, and are mediated in large part by chemokine receptors (such as CCR7) and Rho GTPases [45,46].

Our results indicate that NgR1/2 are up-regulated in the tissue resident cells, and are down-regulated when the cells are activated and required to migrate. This could indicate a possible role for NgR1/2 outside the CNS, perhaps in the activation of DCs or in homing of DCs to specific tissues. This is supported by the findings of the non-myelin associated proteins B cell activating factor (BAFF) and leucine-rich glioma activated (LGI1) as functional ligands for NgR1 [47,48]. BAFF is a TNF-like cytokine that supports survival and differentiation of B cells. It is expressed in many cell types, including monocytes, DCs, neutrophils, stromal cells, activated T cells, B cells, B cell tumours and epithelial cells [49]. Thus, a wide variety of cells have the ability to produce BAFF and might potentially act on immDCs via NgR1.

The possibility that NgR1 plays a functional role in mediating adhesion of DCs to myelin could become important in situations where peripheral immune cells come into contact with myelin debris, such as after neurodegenerative events. Expression of NgR1, TROY and LINGO-1 was found in CD68^+ ^cells (i.e. macrophages, microglia, and a subset of DCs) within chronic, active demyelinating MS lesions and ischemic lesions of acute and old cerebral infarctions [50,51]. DCs are emerging as important players in CNS autoimmunity, specifically in MS [23]. The finding of mature DC markers in the inflamed meninges and perivascular cuffs of active MS lesions has lead to the suggestion that DCs are recruited to and mature within MS lesions [24]. Here, self-antigens are continuously made available by myelin destruction, thus mature DCs can contribute to the local activation and expansion of pathogenic T cells. This model is conducive to our findings of increased adhesion of matDC to myelin, and provides a possible physiological role for the down-regulation of NgR1 in matDC. That is, down-regulation of NgR1 in matDCs promotes their adhesion to myelin, resulting in the selective accumulation of matDCs rather than immDC in the myelin debris-containing lesion. This would result in further antigen presentation and activation of myelin-reactive T cells, potentially aggravating the disease.

## Conclusions

Our study documents the differential expression and function of NgR1 and NgR2 in human DCs. We describe the increased expression of NgR1 and NgR2 in human immature DCs, which are then down-regulated upon maturation. Since human mature DCs adhere to a much higher extent to myelin than immature DCs, we hypothesise that this effect is mediated by NgR1. This finding was corroborated by using mature DCs from *Ngr1/2-/- *mice, which adhere significantly more to a myelin substrate compared to WT mature DCs. The interaction of DCs with myelin provides insight into how DCs act when in the presence of CNS myelin, such as during neurodegeneration and/or neuroinflammation. The down-regulation of myelin-associated inhibitory factor receptors NgR1 and NgR2 on mature DCs may facilitate their initiation of local antigen presentation function during physiological and pathological immune responses in the CNS.

## Competing interests

The authors declare that they have no competing interests.

## Authors' contributions

CMD, KS, CB, and MR conceived and designed the experiments. CB and RS generated and provided *Ngr1/2-/- *mice. CMD and FK carried out all experiments. CMD, KS, FK, RS and MR analysed and interpreted the data. CMD, KS, and MR wrote the manuscript. All authors read and approved the final manuscript.
